# Transarterial Embolization of Geniculate Arteries Reduces Pain and Improves Physical Function in Knee Osteoarthritis—A Prospective Cohort Study

**DOI:** 10.3390/diagnostics14151627

**Published:** 2024-07-27

**Authors:** Louise Hindsø, Per Hölmich, Michael M. Petersen, Michael B. Nielsen, Søren Heerwagen, Mikkel Taudorf, Lars Lönn

**Affiliations:** 1 Department of Radiology, Copenhagen University Hospital Rigshospitalet, Blegdamsvej 9, 2100 Copenhagen, Denmark; mbn@dadlnet.dk (M.B.N.); soeren.heerwagen@regionh.dk (S.H.); mikkel.taudorf@regionh.dk (M.T.); lars.birger.loenn@regionh.dk (L.L.); 2 Faculty of Health and Medical Sciences, University of Copenhagen, Blegdamsvej 3B, 2100 Copenhagen, Denmark; per.hoelmich@regionh.dk (P.H.); michael.moerk.petersen@regionh.dk (M.M.P.); 3 Sports Orthopedic Research Center-Copenhagen (SORC-C), Department of Orthopedic Surgery, Copenhagen University Hospital Amager-Hvidovre, Kettegård Allé 30, 2650 Hvidovre, Denmark; 4 Department of Orthopedic Surgery, Copenhagen University Hospital Rigshospitalet, Blegdamsvej 9, 2100 Copenhagen, Denmark

**Keywords:** trans arterial embolization, genicular artery embolization, embolotherapy, inflammation, knee pain, pain treatment

## Abstract

Knee osteoarthritis (OA) affects millions worldwide, leading to pain and reduced quality of life. Conventional treatments often fail to provide adequate relief, necessitating new therapeutic approaches. This study evaluated the efficacy and safety of genicular artery embolization (GAE) using permanent microspheres in patients with mild-to-moderate knee OA. In this prospective, single-center study, 17 participants underwent GAE. KOOS (Knee injury and Osteoarthritis Outcome Score), WOMAC (The Western Ontario and McMaster Universities Arthritis Index), and IPAQ (International Physical Activity Questionnaire) scores, along with physical performance tests, medication use, and dual-energy X-ray absorptiometry (DEXA) scans, were assessed at baseline and at multiple follow-up points over six months. The primary endpoint, VAS at six months, showed significant improvement (median reduction from 66 mm to 40 mm, *p* = 0.0004). All pain and function scores, as well as physical performance tests, improved significantly. No clinically relevant changes in medication use or DEXA parameters were observed after six months. Only minor, self-limiting adverse events occurred. This study indicates that GAE is a promising minimally invasive treatment for knee OA, providing significant pain relief and functional improvement. However, further long-term, randomized trials are needed to confirm these findings and establish optimal patient selection criteria.

## 1. Introduction

Knee osteoarthritis (OA) is a widespread condition affecting millions of people worldwide [1,2]. Standard treatments include analgesic and anti-inflammatory medications, physical therapy, joint injections, and arthroplasty as an end-stage solution [3]. Patients often experience insufficient pain relief with non-surgical conventional regimens, leading to reduced quality of life and significant socioeconomic burdens [1,2].

Knee OA is a multifactorial disease that often involves chronic inflammation and angiogenesis, which stimulate each other in a positive feedback loop, and collectively contribute to pain and joint destruction [4,5]. The pioneering study by Okuno et al. [6] described genicular artery embolization (GAE) as a minimally invasive endovascular procedure designed to occlude pathological neovessels formed during the progression of knee OA. The trial demonstrated significant pain relief and laid the groundwork for further research in this area.

Published studies on GAE have demonstrated promising efficacy and safety results [7,8]. However, because of the limited number of studies and the heterogeneity in methods and outcomes, it is still too early to draw definitive conclusions. Researching OA is challenging due to the subjective nature of pain, its primary symptom; this underscores the need for including objective outcomes.

The aim of this prospective study was to assess the efficacy and safety of GAE using permanent microspheres in treating mild-to-moderate knee OA by incorporating both patient-reported outcome measures (PROMs), physical performance tests and dual-energy X-ray absorptiometry (DEXA).

## 2. Materials and Methods

This interventional, prospective, single-center, single-arm study was registered and updated at ClinicalTrials.gov (Identifier: NCT05360329, accessed on 19 December 2023). Ethical approval was obtained from the local Ethical Committee (Identifier: H-20081451). The final follow-up was six months.

### 2.1. Participants

Participants were recruited among patients followed for knee osteoarthritis at the orthopedic surgery department. Inclusion criteria were moderate-to-severe knee pain, defined as Visual Analog Scale (VAS) > 50 mm during walking, including walking on stairs, and X-ray-verified mild-to-moderate knee osteoarthritis (Kellgren–Lawrence grade 1–3 [9]). Knee pain had to be resistant to at least 3 months of physiotherapy. The minimum age required was 30 years.

Exclusion criteria were BMI > 35 kg/m^2^; ipsilateral intra-articular knee injection or arthroscopy within six months of study enrollment; prior ipsilateral open knee surgery; local infection in knee or groin areas; moderate-to-severe pain in other ipsilateral lower limb joints, indicated by VAS > 2; intermittent claudication; generalized pain syndromes (e.g., fibromyalgia); nerve root compression syndromes; rheumatoid arthritis; seronegative arthropathies; current or recent (within 4 weeks) use of oral corticosteroids; pregnancy or lactation; active malignancy; contraindications for MRI scan including contrast media allergies, diabetes, liver or kidney disease, or estimated glomerular filtration rate < 60 mL/min/1.73 m^2^; INR > 1.4; platelets ≤ 40 × 10^9^/L; antithrombotic treatment except acetylsalicylic acid; diseases affecting the bone metabolism; and American Society of Anesthesiologists classification > 3.

**Figure 1:**
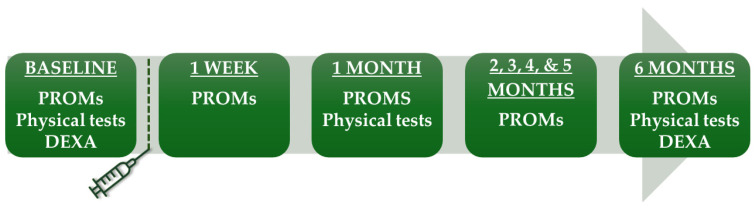
Study flow. PROMs included VAS, KOOS, WOMAC, IPAQ, medication use, and adverse events. DEXA, dual-energy X-ray absorptiometry; IPAQ, International Physical Activity Questionnaire; KOOS, Knee injury and Osteoarthritis Outcome Score; PROMs, patient-reported outcome measures; VAS, Visual Analog Scale; WOMAC, The Western Ontario and McMaster Universities Arthritis Index.

The most painful knee was treated for participants with bilateral knee osteoarthritis. The patient study flow is shown in Figure 1. Baseline visits were conducted two days prior to embolization. All participants signed an informed consent form.

### 2.2. Intervention

**Figure 2:**
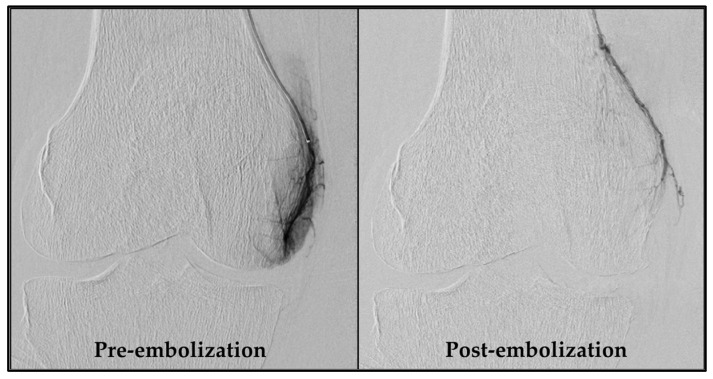
DSA pre- and post-embolization of the descending genicular artery. The **left** image shows a distinct blush due to neovessels, formed as a consequence of chronic inflammation. In the **right** image, these neovessels are pruned by embolization. DSA, digital subtraction angiography.

An experienced interventional radiologist (S.H.) performed all GAE procedures, obtaining femoral artery access with a 4-French sheath via the Seldinger technique after administering local anesthesia in the groin. As a standard, access was obtained through the ipsilateral groin and an antegrade approach. If this was not feasible, access via the contralateral groin area was used. Subsequently, a 4-French C2 catheter (Super Torque, Cordis, Miami Lakes, FL, USA) was introduced, and a digital subtraction angiography (DSA) was performed to map the knee’s vascular anatomy. Vessels supplying painful areas of the knee were selectively catheterized with a guidewire (Fathom 14, straight tip, Boston Scientific, Marlborough, MA, USA) and a microcatheter (1.7-French Carnelian, Tokai Medical Products, Kasugai-city, Japan), as described by Little et al. [10]. Nitroglycerin (100 µg/mL) was initially administered, and subsequently when needed, to prevent spasms. Abnormal neovessels were identified by their angiographic blush-like appearance (Figure 2). Ice packs were applied tightly around the knee to minimize non-target skin embolization. Additionally, a cone-beam CT was performed to check for collaterals. Embolization was performed with 100–300 µm Embosphere^®^ Microspheres (Merit Medical, South Jordan, UT, USA) diluted in 20 mL iodinated contrast (Visipaque, 270 mg/mL, GE Healthcare, Chicago, IL, USA), starting with a 0.2 mL particle suspension and adjusted based on feedback until the neovessels were pruned. Hemostasis at the access site was ensured by manual compression and participants rested for four hours before discharge. Technical success was defined as the embolization of at least one target vessel. Procedure time and radiation dose were recorded.

### 2.3. Outcomes

#### 2.3.1. Patient-Reported Outcome Measures

Participants completed online questionnaires at baseline, one week after embolization, and then monthly until final follow-up at six months (Figure 1). They documented the most severe knee pain experienced during walking, including stair-climbing, in the last 24 h. This was measured using the pain intensity scale VAS, ranging from 0 to 100 mm, from least to most severe pain. The primary endpoint was VAS at six months. In addition, participants filled out the Knee Injury and Osteoarthritis Outcome Score (KOOS [11]) and the short version of the International Physical Activity Questionnaire (IPAQ [12]). From the IPAQ responses, a physical activity score was calculated in MET hours per week (Metabolic Equivalent of Task), a unit that quantifies the energy expenditure of activities [12]. Additionally, the average daily sitting hours were calculated based on the IPAQ responses. The WOMAC score, extracted from the KOOS responses, was transformed to a 0 to 100 scale, from worst to best [11,13]. At the six-month follow-up, participants rated their treatment response on a 5-point Likert scale with the options none, poor, fair, good, and excellent.

#### 2.3.2. Analgesic and Anti-Inflammatory Medication

Participants registered their use of paracetamol, non-steroidal anti-inflammatory drugs (NSAIDs), and opioids at baseline, one week, and monthly post-embolization. No medication was prescribed, controlled, or advised by the study physicians.

#### 2.3.3. Physical Performance Tests

Participants completed physical performance tests at baseline and again at one month and six months post-embolization under the guidance of a supervising physician. These tests included a 40 m fast-paced walk test, a 30 s chair-stand test (seat height 46 cm), and a stair-climb test (13 steps of 18 cm), as recommended by the Osteoarthritis Research Society International (OARSI) guidelines [14].

#### 2.3.4. DEXA Scan

**Figure 3:**
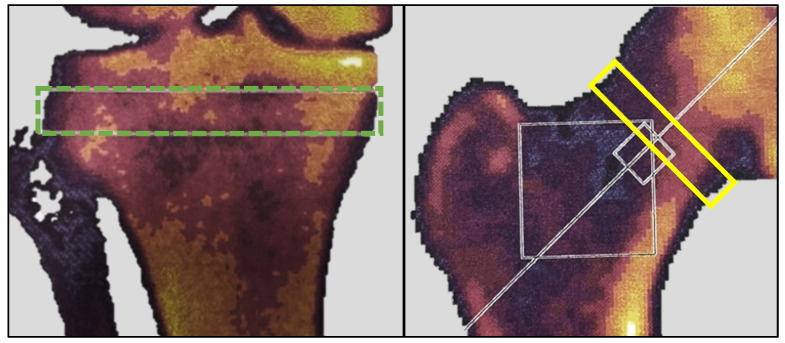
DEXA measurements. The green dotted box represents the area of BMC and BMD measurements. The yellow box represents the area of T-score measurement. BMC, bone mineral content; BMD, bone mineral density; DEXA, dual-energy X-ray absorptiometry.

The rationale for using DEXA in this study was twofold. Firstly, it aimed to measure potential bone mineral changes induced by altered loading of the extremities. The proximal tibia is an anatomical location known to respond rapidly with density changes due to altered loading [15]. Secondly, it sought to describe the study cohort in terms of their general bone mineral status. At baseline and six months post-embolization, all participants underwent a DEXA scan using a Norland XR-46 bone densitometer (Norland Corp, Fort Atkinson, WI, USA). Both hips were scanned at a speed of 90 mm/s with a pixel size of 1.0 × 1.0 mm. The T-score was measured in a 1.5 cm band perpendicular to the femoral neck of the treated leg (Figure 3); for participants with ipsilateral hip implants, measurements were taken from the contralateral hip. Both knees were scanned at a speed of 60 mm/s with a pixel size of 0.5 × 0.5 mm. Bone mineral density (BMD) in g/cm^2^ and bone mineral content (BMC) in grams were measured in the treated knee within a 1 cm band between the fibula head and the subchondral plates (Figure 3), as described by Petersen et al. [16]. The scanner was calibrated and quality-checked daily, and all scans were carried out by a trained research nurse.

### 2.4. Adverse Events

Adverse events were recorded at all follow-up time points, and participants were instructed to contact study physicians for any unexpected occurrences after the final follow-up. Events were graded based on the classification system by the Society of Interventional Radiology [17].

### 2.5. Statistics

Based on a study by Bagla et al. that showed an average VAS reduction of 44 mm (SD 30) [18], we calculated that a sample size of 10 participants would provide 90% power to detect a 50% reduction in VAS at a 5% significance level. We included 20 participants to allow for comprehensive analyses and to account for potential dropouts. Due to the sample size, all continuous variables were reported as medians and interquartile ranges (IQRs). Longitudinal outcomes were analyzed using Friedman’s ANOVA with Dunn’s post hoc analysis and Bonferroni correction. For variables with only one follow-up point, Wilcoxon signed-rank tests were performed. Significance was set at *p* < 0.05. Statistical analyses and graphical representations were performed using SAS Enterprise Guide 7.1, Microsoft Excel Version 2406, and GraphPad Prism 10.

As Supplementary Material (Table S1), we present the means and standard deviations (SDs) for all continuous outcomes, as well as the mean differences and SDs between the 6-month follow-up and baseline. These data might be useful for future meta-analyses and power calculations.

## 3. Results

Of the 20 participants referred for GAE, 17 exhibited significant hyperemic blush during pre-embolization DSA and were successfully embolized. In all these cases, the blush was absent in the post-embolization DSA. Two participants showed no significant blush initially, and one patient experienced localized vessel spasm that impeded distal canalization and subsequently hindered embolization. This resulted in a technical success rate of 85% for the procedure.

On average, 1.3 vessels were embolized per patient. The median procedure time was 135 [IQR 105–157] min, and fluoroscopy time was 28 [IQR 19–42] min. Median radiation dose was 385 [IQR 249–584] mGy, and the dose area product (DAP) was 105 [IQR 53–339] Gy/cm^2^. Of the 17 treated participants, 15 (88%) were treated only on the medial side of the knee, while 2 (12%) received treatment on both the medial and lateral sides.

The 17 treated participants were followed for 6 months, with no loss to follow-up. Baseline characteristics are shown in Table 1.

### 3.1. Patient-Reported Outcome Measures

**Figure 4:**
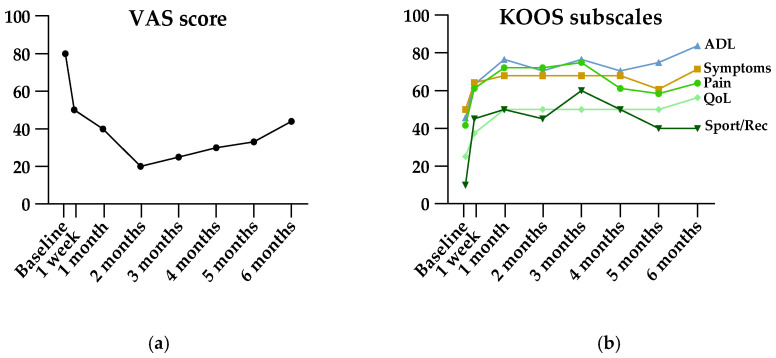
Visual illustration of VAS (**a**) and KOOS (**b**) scores pre- and post-embolization. Data are presented as medians. ADL, activities of daily living; KOOS, Knee injury and Osteoarthritis Outcome Score; QoL, quality of life; Sport/Rec, sport and recreation function; VAS, Visual Analog Scale.

Results of patient-reported measures are shown in Table 2. The primary endpoint, VAS at 6 months, showed a significant improvement from baseline (median 66 mm [IQR 61–73] at baseline, median 40 mm [IQR 20–60] at 6 months, *p* = 0.0004). Friedman’s ANOVA revealed significant improvement in VAS, all KOOS subscales, and all WOMAC subscales (Table 2). As illustrated in Figure 4, VAS and KOOS subscales for pain and sport/recreation function showed decreasing efficacy starting three months after treatment. However, post hoc analysis showed ongoing significant efficacy at the end of follow-up at six months.

**Figure 5:**
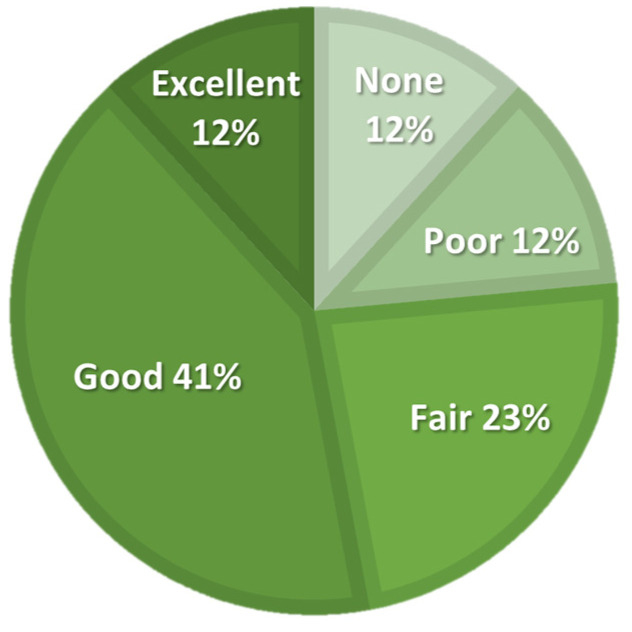
Patient-reported treatment efficacy on a 5-point Likert scale at six-month follow-up.

The activity level, expressed in IPAQ-derived MET hours per week, improved at all follow-up points compared to baseline, but Friedman’s ANOVA did not show significant differences. The number of daily hours spent sitting varied slightly and showed a significant ANOVA result; however, post hoc analysis did not reveal any significant differences between any follow-up points and baseline. The participants assessed the efficacy of the treatment at a 5-point Likert scale, as illustrated in Figure 5.

### 3.2. Analgesic and Anti-Inflammatory Medication

During the study period, minor but not statistically significant changes in medication usage were observed. At baseline, 10 (59%) participants reported paracetamol usage during the last week; this increased to 11 (65%) one-week post-treatment but then dropped to 8 (47%) from one month to the final follow-up. For NSAIDs, eight (47%) participants reported usage at baseline, which increased to nine (53%) one-week post-treatment and then decreased to seven (41%) from one month and for the remaining follow-up period. Additionally, at baseline and from one to five months, one (6%) participant reported use of morphine, while this increased to two (12%) at one-week and again at six months post-treatment.

### 3.3. Physical Performance Tests

**Figure 6:**
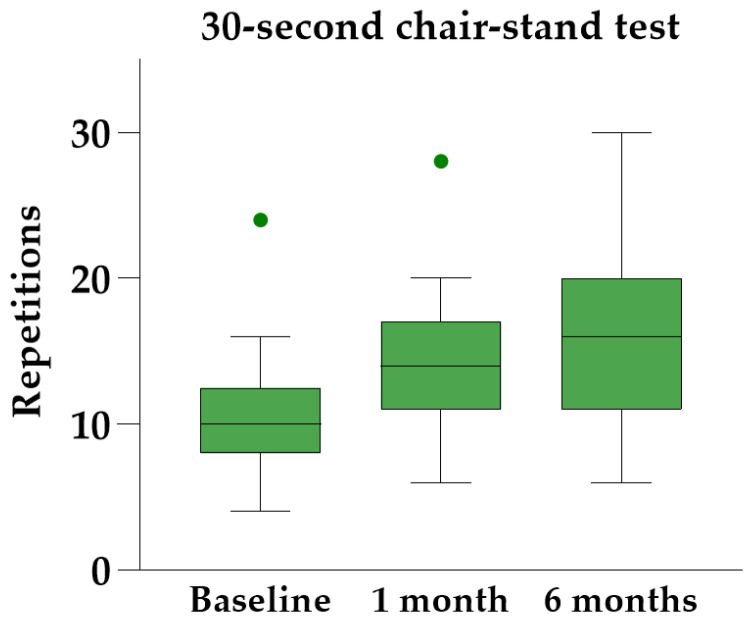
The 30 s chair-stand test at baseline and follow-up, illustrated with Tukey boxplots.

Friedman’s ANOVA with Dunn’s post hoc analysis revealed significant improvements in all three physical performance tests at both follow-up visits compared to baseline (Table 3). The 30 s chair-stand test yielded the most significant results, showing a median individual improvement of 45% [IQR 34–83%] at the six-month follow-up (Figure 6).

### 3.4. DEXA Scan

For the treated knee, the median BMC was 5.8 g [IQR 4.7–7.3] at baseline and 5.8 g [IQR 4.0–7.2] at six months (*p* = 0.056), while the median BMD was 0.80 g/cm^2^ [IQR 0.67–0.92] at baseline and 0.78 g/cm^2^ [IQR 0.60–0.92] at six months (*p* = 0.045). For the untreated knee, BMC was 6.5 g [IQR 4.8–7.2] at baseline and 5.8 g [IQR 4.6–7.4] at six months (*p* = 0.17), while BMD was 0.86 g/cm^2^ [IQR 0.75–0.94] at baseline and 0.80 g/cm^2^ [IQR 0.65–0.96] at six months (*p* = 0.089). The difference in baseline BMC and BMD of treated vs. non-treated knee was not statistically significant. Mean T-score at the femoral neck was −0.8 (ranging from −2.6 to 1.4). Due to a hip prosthesis in the treated leg, the T-score was measured on the contralateral hip in one participant.

### 3.5. Adverse Events

Two participants (12%) experienced post-procedural hematomas at the insertion site, each with a diameter of less than 10 cm. Four participants (24%) observed skin color changes in relation to the area of embolization. These minor adverse events resolved spontaneously within 3 months and necessitated no treatment. There were no reports of severe adverse events. Most participants experienced knee pain in the embolized region up to a week after treatment, attributed to ischemic synovium. No additional treatment beyond patient-administered pain killers was necessary. All participants were discharged as planned four hours post-treatment.

## 4. Discussion

This study investigated the efficacy and safety of GAE using permanent microspheres for treating mild-to-moderate knee OA. Our results showed statistically significant improvements in VAS, KOOS, WOMAC, and physical performance. The median activity level, measured by IPAQ, rose by 65%, but this result was not statistically significant, potentially due to a type II error. Minor fluctuations were observed in DEXA parameters (BMD and BMC) and the use of analgesic and anti-inflammatory medication; however, all were clinically insignificant. Adverse events were mild and transient, with no reports of severe complications.

All previous GAE studies, including meta-analyses, showed improvement in PROMs [19,20]. Landers et al. observed that efficacy ceased after two years [21], while other studies reported continuous efficacy at the end of follow-up at one to two years [22,23,24,25,26,27]. However, patients with severe OA or meniscal injuries may have poorer outcomes [28,29]. Two RCTs examined GAE efficacy beyond placebo [30,31]. Landers et al. found no differences between GAE and sham after 12 months, but a sub analysis showed greater improvements in KOOS sport/recreation and KOOS ADL scores for complete embolization of all genicular arteries [30]. Bagla et al. found superior pain relief and functional improvement with GAE over sham treatment, but did not investigate long-term disparities due to crossover at one month [31].

In our study, many participants reported increased pain in the days following GAE, supported by a slight rise in the use of self-administered painkillers during the first week. This underscores the importance of systematic patient education and post-procedural pain management to improve compliance and satisfaction. Previous studies consistently noted decreased painkiller use post-GAE [7,27,31,32,33].

Physical performance tests are described in two publications by Landers et al. [21,30]. Consistent with our results, both studies observed an increase in physical performance, with the greatest improvement seen in the 30 s chair-stand test. However, in the RCT, no significant difference was found between GAE and sham [30]. To date, no studies have used the IPAQ score or comparable measures to assess physical activity levels.

DEXA parameters are indirect measures of the load and use of an extremity, though they also naturally decline with age [16,34,35]. In this study, the median BMC and BMD values showed a slight decrease in both the treated and untreated knees, likely reflecting natural, age-related bone loss. To our knowledge, DEXA has not been described before in relation to GAE studies. Due to the observed increase in physical performance after GAE, it would be of interest to perform DEXA after a longer follow-up period to determine if the treatment can mitigate age-related bone loss.

The most common adverse events reported in GAE studies are transient skin color changes and minor access site hematomas, consistent with our findings. In general, adverse events after GAE are minor and self-resolving, as listed by the Epelboym et al. [19]. Following their publication, Little et al. [23] described one severe event involving deep venous thrombosis 15 days after GAE, attributed to post-treatment immobilization rather than directly to GAE. Sapoval et al. [36] also reported a severe complication with a significant increase in serum creatinine the day after the procedure, which normalized after 13 days. Bagla previously reported two cases of leg paresthesia [18], and recently two studies each reported one similar case, both of which resolved spontaneously within one month [37,38].

The observed improvements in pain, function, and physical performance highlight GAE’s potential as a minimally invasive treatment for knee OA. Our study not only supports previous findings, but it also uniquely includes physical performance tests, scoring of physical activity levels, and DEXA scans.

Despite promising results, our study has several limitations, including a small sample size and a short follow-up period. Moreover, the lack of a control group limited the evidence of the results. Bilateral knee pain was present in 65% of the participants, which biased the function-related outcomes, particularly the physical performance tests. With a larger sample size, a subgroup analysis of patients with unilateral knee pain might have shown even greater improvement.

GAE reduces inflammation and associated pain by closing the neovasculature [22]. Since the underlying disease is not treated, symptoms are likely to return over time, making retreatment a potential option [21,22]. The potential for GAE to reduce joint destruction by decreasing inflammation, along with the possibility that it could also accelerate joint destruction, requires further investigation. Additionally, the benefits of a pain-free window for increasing physical activity need to be explored. Future research should focus on objective outcomes such as imaging and inflammatory biomarkers, and account for the placebo effect through randomized clinical trials with a sham procedure. Additionally, optimal patient selection, treatment protocols, and pathophysiological research on different embolic materials should be addressed. Moreover, comparisons with standard treatments, evaluation of long-term efficacy and safety, and cost-effectiveness analyses will enhance our understanding of GAE’s clinical utility in knee OA management.

## 5. Conclusions

In this study, GAE with permanent particles proved effective and safe for mild-to-moderate knee osteoarthritis. Significant improvements were seen in PROMs and physical performance tests. Further validation through long-term randomized clinical trials is warranted.

## Figures and Tables

**Table 1:** Baseline characteristics.

	*n* = 17
Women/men, n (%)	9 (53%)/8 (47%)
Age, years, median (range)	56 (43–71)
BMI, kg/m^2^, median (range)	27 (20–35)
KL grade, n (%)	
1	2 (12%)
2	7 (41%)
3	8 (47%)
Bilateral knee pain, n (%)	11 (65%)
Embolized knee, right/left, n (%)	10 (59%)/7 (41%)

BMI, body mass index; KL, Kellgren–Lawrence.

**Table 2:** Patient-reported outcome measures.

	Baseline	1 Week	1 Month	2 Months	3 Months	4 Months	5 Months	6 Months	Friedman’s ANOVA	
**VAS 0–100**	66 [61–73]	55 [39–70]	28 [15–65]	27 [14–50]	20 [10–50]	24 [18–65]	35 [20–65]	40 [20–60]	X^2^ (7) = 42.68	*p* < 0.0001
**KOOS 0–100**										
Pain	42 [36–47]	61 [50–67]	72 [44–81]	72 [50–81]	75 [44–83]	61 [53–78]	58 [53–78]	64 [56–83]	X^2^ (7) = 33.66	*p* < 0.0001
Symptoms	50 [43–68]	64 [54–75]	68 [61–82]	68 [54–79]	68 [54–82]	68 [57–75]	61 [54–79]	71 [61–75]	X^2^ (7) = 15.01	*p* = 0.0358
ADL	46 [41–59]	63 [53–76]	76 [50–91]	71 [60–90]	76 [59–93]	71 [53–93]	75 [62–85]	84 [66–85]	X^2^ (7) = 29.31	*p* = 0.0001
Sport/Recreation	10 [0–25]	45 [25–55]	50 [20–75]	45 [25–70]	60 [25–75]	50 [15–70]	40 [15–70]	40 [15–65]	X^2^ (7) = 29.04	*p* = 0.0001
Quality of Life	25 [19–38]	38 [31–56]	50 [38–56]	50 [44–63]	50 [38–63]	50 [38–69]	50 [38–56]	56 [38–63]	X^2^ (7) = 45.86	*p* < 0.0001
**WOMAC 0–100**										
Pain	50 [35–60]	65 [50–70]	75 [50–85]	75 [50–85]	75 [50–90]	70 [55–85]	70 [50–80]	70 [55–90]	X^2^ (7) = 34.38	*p* < 0.0001
Stiffness	50 [38–75]	63 [50–75]	63 [50–75]	75 [50–75]	75 [50–88]	63 [63–75]	75 [50–75]	75 [50–88]	X^2^ (7) = 21.26	*p* = 0.0034
Function	46 [41–59]	63 [53–76]	76 [50–91]	71 [60–90]	76 [59–93]	71 [53–93]	75 [62–85]	84 [66–85]	X^2^ (7) = 29.36	*p* = 0.0001
**IPAQ**										
MET hours/week	40 [27–123]	47 [21–95]	46 [23–69]	61 [22–108]	72 [49–137]	63 [22–92]	81 [27–179]	65 [41–100]	X^2^ (7) = 11.33	*p* = 0.1247
Hours sitting/day	6 [5–10]	8 [7–10]	5 [4–8]	6 [4–9]	6 [4–8]	7 [5–8]	5 [4–8.5]	6 [5–8]	X^2^ (7) = 14.53	*p* = 0.0425

Variables are presented as medians and interquartile ranges. KOOS and WOMAC 0 to 100, worst to best score. VAS 0 to 100, best to worst score. ADL, activities of daily living; IPAQ, International Physical Activity Questionnaire; KOOS, Knee injury and Osteoarthritis Outcome Score; MET, Metabolic Equivalent of Task; VAS, Visual Analog Scale; WOMAC, The Western Ontario and McMaster Universities Arthritis Index.

**Table 3:** Physical performance tests.

Test	Baseline	1 Month	6 Months	Friedman’s ANOVA
Stair-climb test, s	17 [12–21]	11 [9–16]	11 [8–14]	X^2^ (2) = 26.55, *p* < 0.0001
40 m fast-paced walk test, m/s	1.5 [1.5–1.8]	1.8 [1.6–2.1]	1.9 [1.6–2.1]	X^2^ (2) = 22.53, *p* < 0.0001
30 s chair-stand test, repeats	10 [8–13]	14 [11–17]	16 [11–20]	X^2^ (2) = 27.28, *p* < 0.0001

Data are presented as medians and interquartile ranges.

## Data Availability

The datasets used and/or analyzed during the current study are available from the corresponding author on reasonable request.

## Supplementary Materials

The following supporting information can be downloaded at https://www.mdpi.com/article/10.3390/diagnostics14151627/s1; Table S1: Outcomes reported as mean and standard deviation.
