# Supplementary material for: Transarterial Embolization of Geniculate Arteries Reduces Pain and Improves Physical Function in Knee Osteoarthritis—A Prospective Cohort Study
Source: Diagnostics (Basel). 2024 Jul 27;14(15):1627. doi: 10.3390/diagnostics14151627 (PMC11311436; doi:10.3390/diagnostics14151627)
Supplement: Supplementary file 1 [file diagnostics-14-01627-s001.zip › diagnostics-3097096-supplementary.pdf]

Table S1. Outcomes reported as mean and standard deviation.

	Baseline	1 week	1 month	2 months	3 months	4 months	5 months	6 months	6 months vs baseline Mean diff (SD)
**VAS 0–100**	71 ± 12	53 ± 22	36 ± 25	35 ± 23	30 ± 25	37 ± 28	41 ± 27	40 ± 27	31 ± 28
**KOOS 0–100**									
Pain	43 ± 17	58 ± 21	64 ± 21	66 ± 19	67 ± 21	64 ± 19	62 ± 20	67 ± 19	24 ± 19
Symptoms	53 ± 20	63 ± 21	66 ± 20	65 ± 20	65 ± 20	70 ± 17	66 ± 18	69 ± 17	16 ± 20
Activities of Daily Living	51 ± 16	64 ± 22	73 ± 19	74 ± 17	74 ± 21	71 ± 19	72 ± 19	75 ± 19	24 ± 19
Sport/Recreation	15 ± 16	41 ± 26	45 ± 31	47 ± 30	49 ± 32	45 ± 33	44 ± 30	42 ± 29	27 ± 28
Quality of Life	28 ± 15	42 ± 22	47 ± 23	53 ± 25	51 ± 22	52 ± 23	49 ± 21	51 ± 23	23 ± 16
**WOMAC 0–100**									
Pain	47 ± 19	62 ± 21	68 ± 22	71 ± 19	71 ± 23	69 ± 19	67 ± 21	71 ± 20	24 ± 21
Stiffness	51 ± 25	60 ± 25	65 ± 23	66 ± 24	71 ± 24	68 ± 21	68 ± 23	69 ± 21	18 ± 24
Function	51 ± 16	65 ± 22	73 ± 19	74 ± 17	74 ± 21	71 ± 19	72 ± 19	75 ± 19	24 ± 19
**IPAQ**									
MET-hours/week	112 ± 161	75 ± 84	110 ± 157	100 ± 130	130 ± 115	87 ± 81	112 ± 101	116 ± 142	4 ± 189
Hours sitting/day	6.9 ± 3.2	8.0 ± 2.9	5.8 ± 2.8	6.2 ± 3.0	6.3 ± 3.2	6.6 ± 2.6	5.8 ± 2.9	6.4 ± 2.3	-0.5 ± 2.4
**Physical tests**									
Stair-climb test, seconds	20 ± 17	-	14 ± 8	-	-	-	-	14 ± 10	-6 ± 7
40m fast-paced walk test, m/s	1.6 ± 0.4	-	1.9 ± 0.4	-	-	-	-	1.9 ± 0.5	0.3 ± 0.2
30s chair-stand test, repetitions	11 ± 4	-	14 ± 5	-	-	-	-	16 ± 7	6 ± 4
**DEXA**									
BMC (treated knee), g	5.9 ± 1.3	-	-	-	-	-	-	5.7 ± 1.6	-0.2 ± 0.7
BMD (treated knee), g/cm^2	0.80 ± 0.14	-	-	-	-	-	-	0.77 ± 0.18	-0.02 ± 0.08

Data presented as mean ± SD. Right collum represent mean ± SD of the difference between 6 months follow-up and baseline. KOOS and WOMAC 0 to 100, worst to best score. VAS 0 to 100, best to worst score.

BMC, Bone Mineral Content; *BMD*, Bone Mineral Density; *DEXA*, Dual Energy X-ray Absorptiometry; *IPAQ*, International Physical Activity Questionnaire; *KOOS*, Knee injury and Osteoarthritis Outcome Score; *MET*, Metabolic Equivalent of Task; *VAS*, Visual Analog Scale; *WOMAC*, The Western Ontario and McMaster Universities Arthritis Index.
